# Taurine and its analogs in neurological disorders: Focus on therapeutic potential and molecular mechanisms

**DOI:** 10.1016/j.redox.2019.101223

**Published:** 2019-05-21

**Authors:** Md. Jakaria, Shofiul Azam, Md. Ezazul Haque, Song-Hee Jo, Md. Sahab Uddin, In-Su Kim, Dong-Kug Choi

**Affiliations:** a Department of Applied Life Sciences and Integrated Bioscience, Graduate School, Konkuk University, Chungju, South Korea; b Department of Pharmacy, Southeast University, Dhaka, Bangladesh; c Department of Integrated Bioscience and Biotechnology, College of Biomedical and Health Sciences, and Research Institute of Inflammatory Diseases (RID), Konkuk University, Chungju, South Korea

**Keywords:** Taurine, Analogs, Therapeutic, Molecular role, Clinical study and Neurological disorders, [Ca^2+^]_i_, Intracellular calcium, 3-NP, Nitropropionic acid, AD, Alzheimer's disease, ATP, Adenosine triphosphate, Aβ, Amyloid beta, BBB, Blood-brain barrier, cGMP, Cyclic guanosine monophosphate, ER, Endoplasmic reticulum, GABA, γ-aminobutyric acid, GDNF, Glial cell line-derived neurotrophic factor, IL, Interleukin, IUGR, Intrauterine growth-restricted, NADH/NAD^+^, Nicotinamide adenine dinucleotide (reduced)/ Nicotinamide adenine dinucleotide (oxidized), NF-κB, Nuclear factor-κB, NMDA, N-methyl-d-aspartate, NOAEL, No Observed Adverse Effect Level, PD, Parkinson's disease, PKC, Protein kinase C, ROS, Reactive oxygen species, SOD, Superoxide dismutase, STAT1/3, Signal transducers and activators of transcription 1/3, STZ, Streptozotocin, TauR, Taurine receptors, TauT, Taurine transporter, TBI, Traumatic brain injury, UPR, Unfolded protein response

## Abstract

Taurine is a sulfur-containing amino acid and known as semi-essential in mammals and is produced chiefly by the liver and kidney. It presents in different organs, including retina, brain, heart and placenta and demonstrates extensive physiological activities within the body. In the several disease models, it attenuates inflammation- and oxidative stress-mediated injuries. Taurine also modulates ER stress, Ca^2+^ homeostasis and neuronal activity at the molecular level as part of its broader roles. Different cellular processes such as energy metabolism, gene expression, osmosis and quality control of protein are regulated by taurine. In addition, taurine displays potential ameliorating effects against different neurological disorders such as neurodegenerative diseases, stroke, epilepsy and diabetic neuropathy and protects against injuries and toxicities of the nervous system. Several findings demonstrate its therapeutic role against neurodevelopmental disorders, including Angelman syndrome, Fragile X syndrome, sleep-wake disorders, neural tube defects and attention-deficit hyperactivity disorder. Considering current biopharmaceutical limitations, developing novel delivery approaches and new derivatives and precursors of taurine may be an attractive option for treating neurological disorders. Herein, we present an overview on the therapeutic potential of taurine against neurological disorders and highlight clinical studies and its molecular mechanistic roles. This article also addresses the neuropharmacological potential of taurine analogs.

## Highlights

• Taurine is a naturally occurring sulfur-containing and semi-essential amino acid found within the body.
• Taurine plays several modulatory and regulatory roles in different physiological processes.
• Taurine shows versatile pharmacological role to ameliorate several neurological disorders.
• Due to limitations in crossing the BBB, taurine depends on other mechanisms for functionality.
• The design of taurine formulations may provide potential drugs/supplements for maintaining health and treating disorders of the CNS.

## Graphical abstract

**Figure:**
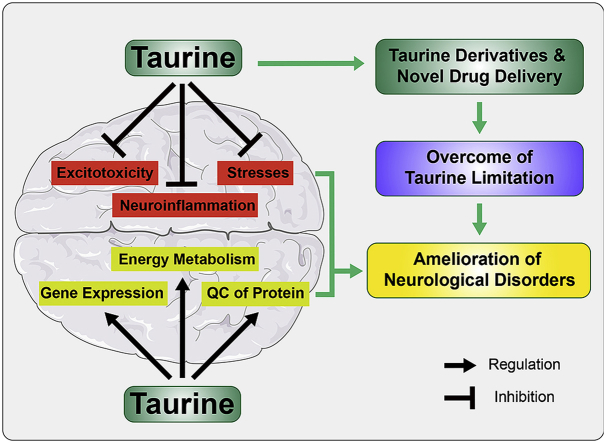

## 1 Introduction

Taurine was first revealed as a constituent of ox bile in 1827 and is a sulfur-containing semi-essential amino acid available in mammals. It plays a crucial role in the developmental processes [[1], [2], [3]]. Taurine is chiefly produced in the liver and kidney; however, it has been found in most other cells and tissues, including the brain, retina, heart, placenta, leukocytes and muscle [4]. It is a crucial factor in various processes such as brain development, optical and immune systems, osmotic regulation, reproduction, stabilization of membranes, cardiac muscle regulation and inflammation [4,5]. For newborn humans, colostrum is essential for developing the retina and brain, which contain a high concentration of taurine. It is commonly included in infant formula and parenteral solutions [4]. As a potential pharmacological agent, its role against oxidative stress and inflammation has been investigated by different studies. It protects against various diseases and disorders in different organ systems such as the integumentary, cardiovascular, respiratory, muscular, skeletal, circulatory and endocrine systems [[6], [7], [8], [9], [10], [11], [12]].

In nervous system disorders, taurine has a broader role, showing protective activity against toxicity in different neurodegenerative disease models for Parkinson's, Alzheimer's and Huntington's diseases [[13], [14], [15]]. Molecular investigations have shown that it may be a neuroprotectant against stroke [16]. It reduced oxidative stress-induced neuropathy in a diabetic mouse model by activating antioxidative defense signals [17]. In addition, recent studies have shown the pharmacological potential of taurine against neurodevelopmental disorders. It protected against retinoic acid-mediated neural tube defects in a mouse model and ameliorated hyperactive behavior in spontaneously hypertensive rats [18,19]. In this article, we describe the molecular mechanistic role of taurine in various neurological disorders, mainly focusing on recent advances from pharmacological perspectives. Moreover, this article provides an overview of taurine and discusses the molecular basis of taurine actions, the pharmacological potential of taurine derivatives and the clinical study of taurine in neurological disorders.

## 2 Overview of taurine

Taurine is available in various types of food. However, it can be found at a low amount in dairy products, including cow's milk and ice cream, and at a high quantity in shellfish, particularly mussels, scallops and clams. Taurine can also be detected in high quantities in the dark meat of chickens and turkeys. Interestingly, cooking does not produce a negative effect on the levels of taurine [20]. Taurine is chemically known as 2-aminoethanesulfonic acid [21]. Humans and cats have limitations in sufficiently synthesizing taurine; however, it is considered the most plentiful free amino acid in mammals [22]. Biosynthesis of taurine occurs in the liver, which initiates from methionine via cysteine, yielding cysteine-sulfonic acid, which is transformed to hypotaurine and taurine. In the hippocampus and cerebellum, taurine biosynthesis occurs via altering the amino acid cysteine by sulfinic acid decarboxylase (taurine-synthase and CAD/CSAD) [23,24]. Taurine displays an exclusive physical property compared to other neuroactive amino acids because sulfonic acid, rather than carboxylic acid, is present in the structure of taurine, which makes difficulty crossing the blood-brain barrier (BBB). As a monobasic acid, taurine's solubility in water is very low (10.48 g/100 mL at room temperature). The pKa value of taurine is 1.5, which is more acidic than that of aspartic acid, glycine, β-alanine and γ-aminobutyric acid (GABA), whereas the pKb value is 8.82, which is less basic than that of GABA, glycine and β-alanine. Taurine displays low passive diffusion due to its cyclic conformation with an intramolecular hydrogen bond [22,25].

As a naturally occurring amino acid, taurine should display minimal side effects in the body. According to toxicity studies, it did not produce genotoxic, carcinogenic or teratogenic effects [16,[26], [27], [28]]. However, a few studies reported tolerable limits of taurine considering the No Observed Adverse Effect Level (NOAEL). Taurine (1000 and 2000 mg/kg/day, i.v.) administration for 13 weeks resulted in water consumption and hemosiderin (a denatured ferritin complex that inefficiently offers existing iron when needed) deposition in the lungs [29]. According to Furukawa et al. (1991), the NOAEL of taurine was 500 mg/kg per day [29]. In contrast, Cantafora et al. (1986) reported that taurine (462 mg/kg/day) in guinea pigs for 2 weeks results in fatty infiltration of the liver [30]. A study of risk assessment has indicated that the highest level of taurine is 3 g per day, which did not show adverse effects according to toxicological indications from a review conducted on all related human clinical trials. However, the lowest dose of taurine with adverse effects has not been set [31]. With respect to the opinion of the European Food Safety Authority, the consumption of 1000 mg/kg per day in energy drinks was considered the NOAEL [16].

## 3 Molecular basis of taurine action against neurological disorders

### 3.1 Modulation of neurogenesis

In the developing brain, taurine has action in neural stem/progenitor cell proliferation [32,33] during which extracellular signal-regulated kinase (ERK)1/2 pathways may be connected to the development of synapse. Taurine influences the levels of proteins such as synapsin 1 and postsynaptic density protein-95, which are crucial in the development of synapses [32]. In another study, it demonstrated a direct action on the proliferation of stem/progenitor cells. Indeed, taurine elevated newborn neuron survival, resulting in improved neurogenesis in the adult [34]. In addition, a recent study demonstrated its antidepressant activity, which may be connected to regulating the hypothalamic-pituitary-adrenal axis and promoting the genesis, survival and growth of neurons in the hippocampus [35].

### 3.2 Modulation of neuroinflammation

The anti-neuroinflammatory activity of taurine has been described by several studies. Taurine significantly increased functional recovery and decreased glial fibrillary acidic protein accumulation and water content in the penumbral region after induced traumatic brain injury (TBI). It significantly prevented growth-related oncogene and interleukin (IL)-1β levels whereas elevating the levels of regulated on activation, normal T cell-expressed and -secreted (RANTES) in comparison with the TBI group. Moreover, a one week treatment with taurine noticeably reduced levels of 17 cytokines, IL-1α, IL-1β, IL-4, IL-5, IL-6, IL-10, IL-12p70, IL-13, IL-17, tumor necrosis factor (TNF)-α, interferon-gamma, eotaxin, granulocyte colony-stimulating factor, granulocyte-macrophage colony-stimulating factor, leptin, monocyte chemotactic protein-1, and vascular endothelial growth factor (VEGF), while elevating the level of macrophage inflammatory protein 1 alpha only. The treatment with taurine effectually reversed brain injury severity in TBI by ameliorating brain edema, elevated activity of astrocytes and proinflammatory cytokines [36]. Taurine (50 mg/kg) efficiently ameliorated pathological inflammation and injury in white matter after intracerebral hemorrhage, and upregulation of H_2_S content and reduction of P2X purinoceptor 7 expression may be connected to those effects [37]. It also ameliorated the accumulation of α-synuclein in paraquat- and maneb-intoxicated mice. In addition, microglial activation caused by paraquat and maneb intoxication was repressed by taurine treatment. Microglial depletion abrogated the neuroprotection of taurine in the dopaminergic system. Afterward, taurine prevented paraquat- and maneb-induced microglial M1 polarization and proinflammatory mediator expression. To initiate and maintain the M1 microglial inflammatory response, the p47phox and nuclear factor-κB (NF-κB) pathways are crucial. Taurine treatment inhibited the activation of NADPH oxidase by affecting both factors. Overall, taurine displayed dopaminergic neuroprotection via inactivating microglia-dependent inflammation in the CNS [14].

### 3.3 Modulation of endoplasmic reticulum stress

**Fig. 1:**
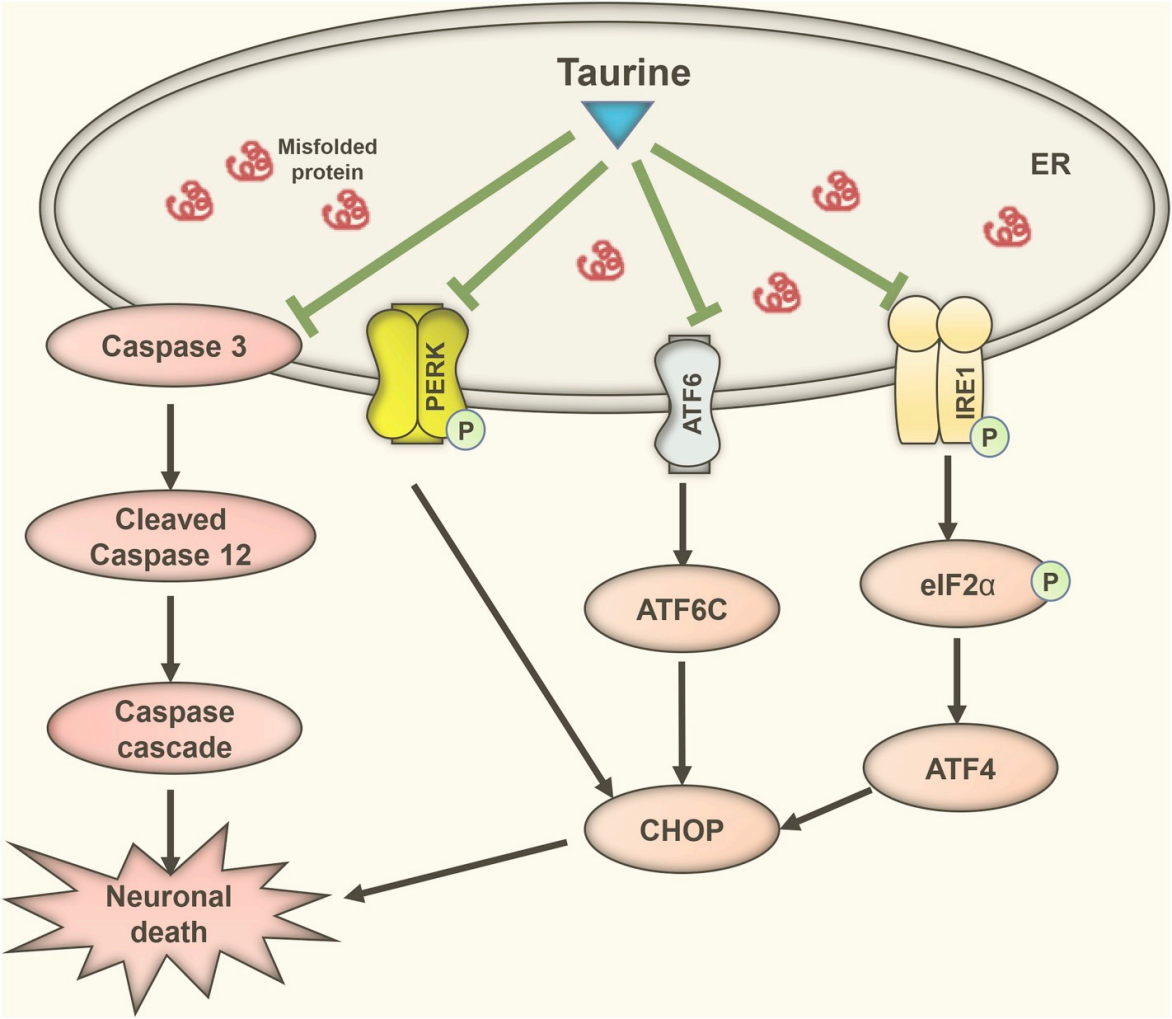
Neuroprotective effects of taurine against ER stress pathways. During ER stress, three types of ER membrane receptors (PERK, ATF6 and IRE1) located at the ER membrane are activated. Phosphorylated PERK converts eIF2α to phosphorylated eIF2α, which activates ATF4, which translocates to the nucleus and transcribes CHOP. ATF6 is converted to its active form, cleaved ATF6, which also transcribes CHOP. IRE1 is activated by phosphorylation and ultimately potentiates the expression of CHOP. Caspase-12, located on the outer surface of the ER membrane, is cleaved into its active form, which facilitates caspase cascade apoptosis. Taurine exerts neuroprotective effects by inhibiting the activation of PERK, ATF6, IRE1, and caspase-12.

As a regulatory mechanism, endoplasmic reticulum (ER) stress is crucial for restoring ER function and re-establishing an equilibrium between protein degradation and protein biosynthesis/folding. Excessive ER stress-stimulated cellular pathways leading to cell death. A well-known originator of ER stress is the gathering of faulty proteins, whose levels rise as a consequence of unsuitable protein folding, insufficient protein degradation or dysfunction of the ER. Three different stress sensors, protein kinase RNA-like endoplasmic reticulum kinase (PERK), activating transcription factor 6 (ATF6) and inositol requiring enzyme-1 (IRE1), are activated by unfolded or misfolded proteins. Upon activation, they recruit the unfolded protein response (UPR) pathways and ultimately work to reestablish ER function and balance between protein degradation and protein biosynthesis/folding. Collectively, the UPR pathways are effective for halting protein biosynthesis, enriching protein degradation, producing chaperones to recover protein folding and triggering either autophagy or apoptosis. In a stroke model, taurine reduced glutamate-mediated toxicity by reducing oxidative stress and an overload of [Ca^2+^]_i_. It also blocked two of the three UPR pathways. Although the mechanisms underlying taurine's action against ER stress and UPR pathways remain to be explored, it is known that taurine deficiency is connected to the ER stress [38,39]. ER stress, oxidative stress and dysfunction in mitochondria are distinguishing states of stroke and several neurodegenerative diseases. A substantial quantity of glutamate is released during a stroke, which overstimulates postsynaptic neurons and causes a neuroexcitotoxic response induced by oxidative stress, [Ca^2+^]_i_ overload, ER stress and, in certain cases, cell death [38,40]. In cellular and animal models of stroke, the action of taurine against ER stress has been described by early studies [41,42]. The effect of taurine against ER stress to protect neuronal damage is presented in Fig. 1.

### 3.4 Modulation of apoptosis

Taurine has modulatory activity on apoptosis as shown by several investigations. It has been considered a potential marker of apoptosis in gliomas [43]. Taurine treatment (20–160 mM) shows a significant preventive action on cell proliferation while promoting the induction of hepatocellular carcinoma HepG2 cell apoptosis [44]. It can inhibit the proliferation of human lung cancer cell line A549 and the growth of transplanted tumors in nude mice. Taurine also promotes the apoptosis in A549 cells by increasing the protein level of p53 upregulated modulator of apoptosis (PUMA) and bcl-2-like protein 4 (Bax) and decreasing the protein level of B-cell lymphoma 2 (Bcl-2). In nude mice-transplanted tumors, PUMA plays a critical role in the taurine action against lung cancer and may signify a novel target for gene therapy in lung cancer [45].Taurine prevented nitric oxide-induced apoptotic cell death in murine RAW264.7 macrophages [46] and myocardial ischemia-induced apoptosis by preventing the assembly of the Apaf-1/caspase-9 apoptosome [47]. Taurine displays neuroprotective activity against hypoxia-induced injury in rats by modulating apoptotic damage. Treatment with taurine (30 mg/kg, i.p., 18 days) increased Bcl-2 expression but reduced Bax and caspase-3 expression [48]. A recent study suggests that taurine is a cytoprotective nutrient that ensures normal mitochondrial and ER function, which is crucial for reducing risk of apoptosis and premature cell death [49]. In addition, the anti-apoptotic role of taurine was investigated in a very recent study in a traumatic brain injury model. Taurine treatment may be effective against apoptosis-mediated brain injury. In addition to anti-apoptotic activity, it demonstrated action against inflammation and oxidative stress in injured brain cells [50].

### 3.5 Role in energy metabolism

The deficiency of taurine causes diminished complex I activity and disturbs energy metabolism mainly by elevating the NADH/NAD^+^ ratio, which controls energy metabolism by feedback inhibition of crucial dehydrogenases. To increase the NADH/NAD^+^ ratio, the citric acid cycle is very sensitive. Three NADH-sensitive enzymes such as α-ketoglutarate dehydrogenase, isocitrate dehydrogenase and citrate synthase, are subject to inactivation by increasing the ratio between NADH and NAD^+^. For instance, pyruvate oxidation in the taurine-deficient heart decreases, as an increased NADH/NAD^+^ ratio stops the activity of pyruvate dehydrogenase and results in a deficiency in pyruvate due to the substantial conversion of pyruvate to lactate [38,51]. Thus, despite stimulated glycolysis, glucose oxidation is noticeably declined in taurine-deficient hearts, which intensely reduces the contribution of glucose metabolism toward overall adenosine triphosphate (ATP) biosynthesis. The taurine biosynthesis rate in the liver is low in humans, so diet is the major source of taurine for humans. According to the study of Jeejeebhoy et al. (2002) [38,52], taurine may be a therapeutic agent for cardiovascular disease because deficiency of taurine has been found in patients with heart failure. Providing taurine supplements restored taurine levels in these patients and resulted in improved contractile function. Numerous proteins and enzymes in fatty acid metabolism, with the most crucial being the long chain fatty acyl carnitine transporter complex, are regulated by PPARα [38,51]. Although the substantial catabolism occurs in the oxidation of fatty acids, it decreases in part because of a reduction in citric acid cycle flux. Additionally, averting fatty acid oxidation during taurine deficiency is due to a low level of PPARα [38,51].

### 3.6 Regulatory role in gene and protein expressions

Taurine triggers genetic changes investigated first time by Park et al. (2006) [53]. Four enzymes such as branched-chain amino acid aminotransferases 2, mitochondrial, branched-chain aminotransferase 1, cytosolic, branched-chain keto acid dehydrogenase, and 3-hydroxy-3-methylglutaryl-CoA lyase are upregulated by taurine. These enzymes are involved in branched-chain amino acid catabolism [53]. Lombardini (1992) reported inhibitory actions of taurine on the phosphorylation of specific proteins in the brain, retina and heart [54]. It is also stated that taurine modifies the phosphorylation of protein [55,56]. The systolic and diastolic defects are seen in the taurine deficit hearts that is linked to decreased sarcoplasmic reticular Ca^2+^ ATPase activity, a modification arbitrated in part by decreased phospholamban phosphorylation [56]. The depletion of taurine accelerates aging by decreasing longevity and accelerating aging-associated tissue damage; these properties probably connected to a disturbance in protein folding [57]. Tissue taurine depletion in taurine transporter (TauT) knockout mouse was found to shorten lifespan and accelerate skeletal muscle histological and functional defects such as an elevation in central nuclei containing myotubes, a reduction in mitochondrial complex 1 activity and induction in cyclin-dependent kinase 4 inhibitor A (an aging biomarker). In addition, tissue taurine depletion increases UPR, which may be connected with an enhancement in protein folding by taurine [57]. Transcription factor, PPARα, content is also modulated by taurine. The level of PPARα is reduced in the taurine deficient heart [51].

### 3.7 Role in neuromodulation

Upon binding to specific taurine receptors (TauR), taurine promoted neuronal hyperpolarization through the opening of chloride channels [58,59]. In addition, acting specifically on GABA_A_, GABA_B_ and/or the glycine receptor, it produced depressive activity [60]. The activity of taurine on GABA_A_ receptors counters seizures mediated by a GABA_A_ antagonist (picrotoxin) via elevating the latency of seizures [61]. Indeed, the inhibitory activity of taurine on the GABA_A_ receptor elevates the level of GABA and enhances the production of glutamic acid decarboxylase isoforms such as GAD 65 and 67, which are connected to GABA synthesis [62].

Taurine-mediated calcium homeostasis regulation is connected to its protective action against excitotoxicity that results from glutamate mediation. An influx of Ca^2+^are resulted due to the affinity of glutamate to N-methyl-d-aspartate (NMDA) receptors, which triggers a cyclic guanosine monophosphate (cGMP)-mediated pathway terminating in protein kinase C (PKC) activation, resulting to decreasing magnesium blockage of NMDA channels and elevating Ca^2+^ influx and excitotoxicity [63]. The triggering of neurotoxicity is activated in a stress situation via an excess delivery of glutamate, and taurine is rapidly recruited for delivery in this state [64]. In addition, it has been shown that neuroprotection by taurine against amyloid beta (Aβ) and glutamate receptor agonists may involve neutralizing NMDA receptors and reducing glutamate delivery and nitric oxide superproduction through GABA_A_ activation. These effects suggested a pharmacological potential of taurine against neurological disorders [65]. Similar results were shown of taurine's activity against ammonia accumulation and cerebral edema, which accounts for its neuroprotective activity [66,67].

Concentrations of taurine are reliant on feeding and taurine's complex transport across TauT at the BBB, which can direct preservation of taurine concentrations in the brain to defend it against the CNS damage [68].

### 3.8 Regulatory role in quality control processes

The ubiquitin-proteasome system and autophagy are regulated by taurine as a part of its role in regulating quality control processes. There are two roles involved in these processes: they revitalize injured cells and subcellular organelles or eradicate them via degradation or cell death. Cells lack taurine, and a decrease in the action of the proteasome leads to an accumulation of ubiquitinated proteins, a consequence eradicated by mitoTEMPO, which is a specific mitochondrial antioxidant [38,69]. Taurine deficiency is also linked to diminished autophagy, which permits the buildup of damaged cells and organelles [69]. Inactivating these quality control processes are very harmful to cells and tissues. Though unwarranted autophagy is also harmful and can cause cell damage. Some studies have investigated the activity of taurine administration on autophagy during which the action displayed by taurine is compatible with its action for cytoprotection, as it ameliorated toxin-mediated autophagy [70,71].

### 3.9 Role in Ca^2+^ homeostasis modulation

Taurine modulates the Ca^2+^ homeostasis that studied by several pieces of research [38]. During myocardial infarction or stroke, Ca^2+^ accumulates extremely within the heart and brain, which is toxic to cells. High [Ca^2+^]_i_ activates lipases and proteases and causes the transition of mitochondrial permeability, an incident that permeabilizes the inner mitochondrial membrane and promotes the pro-apoptotic factor release from mitochondria that ultimately destroy the cell [72,73]. The cytoprotective role of taurine has been demonstrated via diminishing Ca^2+^ overload during which three mechanisms were involved. Firstly, ischemia-reperfusion insult causes loss of taurine at the cellular level, which is arbitrated by the TauT. As the loss of taurine is also accompanied by the loss of Na^+^ from the cell, upon taurine release, less Na^+^ is accessible for Ca^2+^ entry via the Na^+^/Ca^2+^ exchanger, which reduces the degree of Ca^2+^ overload [74]. Secondly, taurine indirectly controls the action of the sarcoplasmic reticular Ca^2+^ ATPase, which is accountable for the maintenance of cytosolic Ca^2+^ homeostasis via the elimination of Ca^2+^ from the cytosol [56]. Thirdly, treatment with taurine is connected to changes in the occurrence of calretinin, calbindin D28k and parvalbumin [75]. Most importantly, taurine protects glutamate-induced influx of Ca^2+^ via the L-, P/Q- and N-type voltage-gated Ca^2+^ channels, along with the NMDA receptor channel [76].

### 3.10 Osmotic regulation

In response to an increase in osmotic stress, the intracellular level of taurine is elevated, while it reduces stress due to hypo-osmosis. These are crucial mechanisms to defend cells from extreme stretching in response to osmotic inequalities. As an organic osmolyte, taurine modifies different osmolyte levels such as the Na^+^ level. Na^+^ has numerous dynamic functions at the cellular level including transport and membrane potential [38,77].

## 4 Therapeutic potential of taurine against neurological disorders

### 4.1 Role in depression and anxiety

According to some studies, taurine possessed antidepressant and anxiolytic activities. In ethanol-induced CNS depression in rats, different doses (7.5, 14.0 and 25 μmol/kg, i.c.v.) of taurine elevated ethanol-mediated sleep time. Interestingly, taurine's antagonist, 6-aminoethyl-3-methyl-4H-1,2,4-benzothiadiazine-l,l-dioxide hydrochloride, inhibited taurine action [78]. In a molecular study of unpredictable mild stress-induced depressive rats, taurine (200 mg/kg or 500 mg/kg, i.p. for a week) displayed antidepressant activity by protecting the decline in sucrose consumption and prevented the lack of spatial memory and increased anxiety in rats, signifying a defensive outcome of taurine on depression-like performance. Moreover, preadministration of taurine reduced the levels of dopamine, 5-hydroxytryptamine, and noradrenaline and reversed elevated glutamate and corticosterone levels. It also protected fibroblast growth factor-2, vascular endothelial growth factor and brain-derived neurotrophic factor expression, which was reduced in depressive rats [79]. Taurine has a potential effect in a mouse model of anxiety. The administration of taurine supplementation increased locomotor activity, while taurine injection repressed that activity in the open-field test. In addition, taurine supplementation caused anxiety, but taurine injection prevented anxiety [80].

### 4.2 Role in neurodegenerative diseases

Taurine produced pharmacological activities in the model of neurodegenerative disease. In the streptozotocin (STZ)-induced Alzheimer's disease (AD) model, taurine (50 mg/kg, p.o. for 15 days) protected from the depleted content of glutathione (GSH) and elevated level of thiobarbituric acid reactive substances in rats. It also prevented from the depletion of antioxidant enzymes glutathione peroxidase, glutathione reductase, glutathione-S-transferase, catalase, and superoxide dismutase (SOD) and alteration of the morphology of the hippocampal pyramidal neurons compared to the STZ-induced group [81]. In another study, treatment with taurine (200 mg/kg, i.p. for 7 days) protected from the elevated production of age-related lipid peroxidation products [82]. Moreover, taurine (250 mg/kg, p.o. for 10 days) attenuated cognitive deficits by directly binding to oligomeric Aβ in mice [83], and taurine (1000 mg/kg per day for 6 weeks) recovered cognition in the adult APP/PS1 mouse model [84]. The mechanism of taurine action as a cholinergic signal has been described. In subchronic exposure of manganese, taurine ameliorated impaired learning and memory ability [85]. Taurine treatment also restored acetylcholinesterase and choline acetyltransferase activities, which are crucial for acetylcholine regulation in both STZ- and Mn-induced models [81,85]. In addition to the cholinergic signaling pathway, taurine prevented chick retinal neurons in cell culture against Aβ-mediated neurotoxicity and glutamate receptor agonists. Picrotoxin, an antagonist of GABA_A_ receptors, has been blocked by the neuroprotective role of taurine; however, the effect is not arbitrated by glutamate receptors [65].

In addition to the AD model, the neuroprotective action of taurine against Parkinson's disease (PD) has been studied in cellular and animal models. Taurine exerted an ameliorating action against rotenone-induced neurodegeneration [86,87]. It displayed a concentration-dependent reduction in rotenone-induced cell damage in SH-SY5Y cells. The combination of a subeffective dose of taurine and low and subeffective doses of N-acetyl cysteine afforded better cytoprotection against rotenone induction than taurine treatment alone and action may be mediated via anti-oxidative mechanisms [86]. In a rotenone-induced rat model, taurine significantly ameliorated rotenone-induced decreases in the levels of catecholamine neurotransmitters and tyrosine hydroxylase. It also attenuated rotenone-induced catalase and lipid peroxidation levels [87]. In PC12 cells, treatment with taurine produced protection against toxic agent-induced degeneration [[88], [89], [90]]. Taurine also restored reduced Bcl-2 expression in an H_2_O_2_-induced model. It reduced H_2_O_2_-induced upregulation of binding immunoglobulin protein (GRP78), growth arrest and DNA damage 153 (GADD153)/C/EBP homologous protein (CHOP) and Bim, signifying that taurine may also play a preventive role against oxidative stress by decreasing ER stress [88]. Against perfluorooctane sulfonate-induced degeneration, administration of taurine also displayed protective activity in PC12 cells. Taurine reduced reactive oxygen species (ROS) production and attenuated perfluorooctane sulfonate-induced increases in autophagy and apoptosis [89]. Moreover, treatment significantly reversed the decrease in viability, oxidative stress and abnormal autophagy in PC12 cells exposed to BDE 209 [90]. Taurine also exhibited protective activity against MPP^+^-induced neurodegeneration in coronal slices from rat brains. Concentrations of taurine at 1 and 20 mM displayed a potentially protective role in cases of neuronal insult [91]. A recent study described taurine's potential effects against neurodegeneration in a PD model. Taurine protects manganese-induced neuronal injury during the physiological outcome of a cilio-inhibitory dopaminergic system in *Crassostrea virginica* [92]. In a paraquat- and maneb-induced neurotoxicity model of mice, treatment with taurine (150 mg/kg, i.p.) attenuated a paraquat- and maneb-mediated decrease in tyrosine hydroxylase-positive neurons in the locus coeruleus. Taurine ameliorated toxin-induced microglial activation and M1 polarization as well as proinflammatory cytokine release in the brainstem of mice. Treatment with taurine also prevented the activation of microglial NADPH oxidase and oxidative damage in paraquat- and maneb-intoxicated mice. In addition, inhibiting NF-κB, but not signal transducers, and activators of the transcription 1/3 (STAT1/3) signaling pathway contributed to taurine-prevented microglial activation [93].

Apart from AD and PD models, taurine treatment produced neuroprotective activity against 3-nitropropionic acid (3-NP)-mediated neuronal cell death in a Huntington's disease model [94,95]. Pretreatment (200 mg/kg, 3 days) with taurine ameliorated behavioral dysfunctions and increased GABA concentration in comparison with 3-NP-induced animals. Treatment also displayed activity against 3-NP-induced oxidative stress as shown by decreased striatal malondialdehyde and increased striatal GSH levels. Moreover, it significantly increased the activity of succinate dehydrogenase compared to that in 3-NP-administered animals. Taken together, taurine neuroprotection in a current Huntington's disease model is due, at least partially, to its indirect antioxidant activity and GABA agonistic action [94]. In another study, taurine exhibited less glial fibrillary acidic protein, SOD, and taurine immunoreactivity, together with increased survival rates in 3-NP-induced rats [95]. In an amyotrophic lateral sclerosis model, it protected cultured motor neurons from glutamate-induced neurotoxic injury [96]. Taurine protected motor neuron loss in amyotrophic lateral sclerosis transgenic mice, in which heat shock factor 1**-**mediated TauT expression partly defends motor neurons by preventing oxidative stress [97].

### 4.3 Role in stroke

Taurine displays actions against several conditions including neuroinflammation, excitotoxicity, oxidative and ER stresses, and apoptosis [37,47,[98], [99], [100]]. Due to these actions, taurine may be a potential protective agent for treating stroke.

In a rat model of intracerebral hemorrhage, taurine administration displays anti-neuroinflammatory activity and prevents white matter injury. Treatment noticeably reduces neutrophil infiltration, glial activation and inflammatory mediator expression. In addition, taurine treatment increases H2S content and cystathionine-β-synthase expression but reduces P2X7R expression [37]. Taurine protects against glutamate-induced excitotoxicity by regulating [Ca^2+^]_i_ in cultured neurons. The mechanism underlying taurine's action in maintaining [Ca^2+^]_i_ homeostasis is at least partly through its inhibition of [Ca^2+^]_i_ influx by preventing the reverse mode of Na^+^/Ca^2+^ exchangers [98]. In addition, taurine shows protective action against nickel chloride (NiCl_2_)-induced damage in cortical neurons. Treatment with taurine (10 mM) markedly reduced NiCl_2_-mediated lactate dehydrogenase (LDH) release, ROS generation and mitochondrial superoxide concentration. Treatment also ameliorated the 24-h NiCl_2_-induced decrease in SOD action and GSH concentration in neurons. In addition, taurine ameliorated NiCl_2_-mediated declined ATP production, interrupted mitochondrial membrane potential and reduced mtDNA content [101]. A recent study also shows the neuroprotective action of combined taurine and DETC-MeSO in preventing ER stress in a rat stroke model. However, they did not produce action separately, while subcutaneous administration of combined treatment (0.56 mg/kg DETC-MeSO) or 40 mg/kg of taurine diminished infarct size and an enhanced neuroscore (reflecting decreased neurological deficit) in rats with MCAO. In addition, combined treatment prevented the expression of the ER stress markers phospho-PERK, phospho-eukaryotic initiation factor 2 (eIF2) α and cleaved ATF-6 [99]. Subcutaneous administration of taurine (5 g/kg) protects against ethanol-mediated apoptosis in cells in the cerebellum. Taurine treatment prevents caspase-3 activation and DNA fragmentation via resorting Bcl-2, regulating [Ca^2+^]_i_ and preventing caspase-9 activation [102]. In the supraoptic and paraventricular nuclei of the hypothalamus, 20 mM taurine treatment reduced ischemia-mediated caspase-8 and caspase-9 immunoreactivity compared with the untreated group [103]. In another study, taurine combination therapy with tissue plasminogen activator (tPA) may ameliorate a delay in tPA-associated hemorrhagic transformation but extend tPA treatment time. In addition, the defensive mechanism of taurine was demonstrated when it inhibited MMP-9 in brain microvessels via inactivating NF-κB and CD147 signaling [104]. In a recent study, taurine significantly improved neurological function and significantly declined water content in the brain and infarct volume in comparison with the MCAO group. Overexpression of 12/15-lipoxygenase and taurine treatment suggestively abridged the expressions of p38 mitogen-activated protein kinase, calcium-dependent phospholipase A2, TNF-α, IL-6 and IL-1β. Therefore, the preventive role of taurine against cerebral ischemia may occur via inhibiting the 12/15 lipoxygenase pathway in rats [105].

### 4.4 Role in traumatic brain and spinal cord injuries

Taurine plays a potential role against trauma-mediated brain and spinal cord injuries. Taurine (2, 5, 15 and 50 mg/kg, i.v. for 7 days) protected the brain against closed head injury by enhancing neurological functions in injured rats, also decreasing brain edema and permeability of the BBB. Taurine treatment also increased SOD activity and glutathione levels but decreased malondialdehyde and lactic acid levels in traumatized tissue. Taurine treatment also prevented cell death in the hippocampus (CA1 and CA3 subfields) [106]. In another study conducted on TBI, the administration of taurine (200 mg/kg for 7 days) by tail intravenous injection protected against neuronal damage in rats. Mitochondrial electron transport chain complexes I and II displayed greater activity in the taurine-treated group, and taurine treatment in cerebral blood flow may alleviate edema and elevated intracranial pressure [107]. In addition to prior studies, taurine treatment (200 mg/kg for 7 days. i.p.) also alleviated brain damage severity in rats by ameliorating the excited activity of astrocytes and edema along with proinflammatory cytokines [36]. Moreover, taurine (25, 80, 250, and 800 mg/kg, i.p.) treatment ameliorated motor disturbance and pathological anomalies in a mouse model of spinal cord injury (SCI). It suggestively reduced the SCI-mediated increase in the levels of IL-6 and myeloperoxidase in a dose-dependent manner. Additionally, taurine significantly reduced SCI-mediated cyclooxygenase-2 and phosphorylated signal transducer and activator of transcription 3 expression. In addition, taurine treatment reduced neutrophil accumulation exclusively in the subarachnoid spaces and induced secondary degenerative deviations in the gray matter [108].

### 4.5 Role in epilepsy

The potential of taurine against epilepsy has been reported by several studies. However, a study showed that pretreatment with taurine (100 mg/kg, p.o.) failed to decrease pilocarpine-induced oxidative stress during status epilepticus [109]. Interestingly, taurine therapy with green tea extract containing 100 mg/kg epigallocatechin gallate attenuated pilocarpine-induced neuronal damage, likely by preventing oxidative stress, insults of hyperexcitability and excitotoxicity [109]. Taurine (2.6 mg/kg, i.p.) also showed anticonvulsant activity in a 4‐aminopyridine-induced seizure mouse model [110]. Treatment with taurine elevated the latency of clonic seizures but decreased the incidence of tonic seizures and postconvulsive mortality. In addition, treatment with taurine (150 mg/kg, i.p.) produced anti-epileptic activity against kainic acid-induced seizures in mice [111]. It produced agonistic action via the GABA receptor. In the brain, increasing the function of the GABA receptor elevates the antagonistic property within the limbic system, which is linked to the anti-epileptic activity of taurine against kainic acid-induced seizures in mice [112]. These findings are supported by a recent article that showed taurine's anti-epileptic activity against pentylenetetrazole-kindled mice. Although the anti-seizure drugs (ASDs) lamotrigine (15 mg/kg), levetiracetam (40 mg/kg), carbamazepine (40 mg/kg), and phenytoin (35 mg/kg) could not suppress generalized tonic-clonic seizures in pentylenetetrazole-kindled mice, administration of a taurine supplement (50, 100 & 200 mg/kg) with ASDs restored the anti-seizure effect of tested ASDs [113].

### 4.6 Role in diabetic neuropathy

Different studies have demonstrated the pharmacological potential of taurine against diabetic neuropathy [114,115]. Among the various properties, its action against oxidative stress-mediated damage may be involved in an emerging role in diabetic neuropathy models. High glucose-induced oxidative stress in human Schwann cells was ameliorated by taurine treatment. Treatment with 0.25 mM taurine reversed high glucose-induced ROS generation [116]. In another study, six weeks of administration of 1% taurine-supplemented diets prevented STZ-induced oxidative stress and nerve growth factor deficit in rats [117]. The action of taurine against oxidative stress was validated by a further study that was conducted on the STZ-induced model. Treatment with 2% taurine for 8 weeks repressed NF-κB expression but enhanced the expression of nuclear factor (erythroid-derived 2)-like 2, Heme oxygenase 1, glucose transporter 1 and glucose transporter 3. Considering these results, taurine-treated activation of the antioxidant signaling pathway may be connected to its effect against oxidative stress [118]. High glucose treatment reduced Vmax and the expression of TauT at the mRNA and protein levels. TauT expression and kinetics were restored by the aldose reductase inhibitor sorbinil and the antioxidant α-lipoic acid. Reestablishment of TauT expression by those agents was linked to an increase in intracellular sorbitol and nitrosylation or glycation with glucose-mediated TauT downregulation [116]. Apart from oxidative stress, taurine treatment also prevented high glucose-induced increases in 4-hydroxynonenal adducts and poly(ADP-ribosyl)ated proteins [116]. Another study conducted on an experimental model of diabetic neuropathy also showed a link between taurine and neurological impairment. Depletion of taurine in the vascular endothelium and Schwann cells of the sciatic nerve may be related to neurovascular and metabolic impairment [114]. Moreover, the antinociceptive action of taurine also has potential for its therapeutic role against diabetic neuropathy. Taurine (2% supplemented diet for 6–12 weeks) ameliorated STZ-induced hyperalgesia and abnormal calcium signaling in rats [115]. Additionally, spinal administration of taurine (200 μg) significantly ameliorated mechanical allodynia and hyperalgesia in STZ-induced diabetic rats. Different doses of taurine administration reduced motor impairment, and a significant outcome was observed at 400 μg. Pretreatment with the glycine receptor antagonist strychnine entirely blocked the antinociceptive properties of taurine; therefore, taurine-mediated neuropathic pain may be linked to glycinergic neurotransmission [119].

### 4.7 Miscellaneous roles

#### 4.7.1 Arsenic-induced neurotoxicity

Taurine has a potential effect in a metalloid-induced neurotoxicity model. It shows an effect against arsenic-mediated oxidative and nitrosative stresses in mice. Induction of arsenic generated ROS and superoxide radicals and also elevated the lipid peroxidation, protein carbonylation and glutathione disulfide levels. In addition, arsenic significantly reduced the actions of acetylcholinesterase, and anti-oxidant and membrane-bound enzymes. In a rat model, taurine (100 mg/kg for 5 days, p.o.) effectively protected arsenic-induced oxidative damage in the brain tissue [120]. Arsenic caused reactive nitrogen species (RNS) generation, and swelling, manifested as vacuolar degeneration in the cytoplasm, karyorrhexis and karyolysis in brain tissues of mice, but taurine treatment ameliorated relatively mild arsenic-induced pathological changes model. A major product, 8-nitroguanine, is formed by a reaction between guanine and ONOO^−^. A common marker of nucleic acid damage, 8-nitroguanine generated as a consequence of RNS attack. Coadministration of taurine and arsenic caused weak 8-nitroguanine expression in mouse brain cells. However, arsenic induction alone showed intensive 8-nitroguanine expression in mouse brain tissue that mostly spread in the nucleus at the nuclear membrane but was minimal in the cytoplasm [121]. According to both studies, taurine ameliorated arsenic-induced cerebral oxidative and nitrosative stresses and alleviated arsenic-mediated DNA damage in brain neurons via the RNS signal pathway.

#### 4.7.2 Bilirubin-induced neurotoxicity

Regarding study data on neonatal jaundice, taurine may be a potential compound for averting and/or treating neuronal damage. Taurine showed protective activity against unconjugated bilirubin (UCB)-induced damage in primary neuronal cultures. UCB elevated the proportion of cell apoptosis and [Ca^2+^]_i_ levels, but taurine intensely protected against UCB-induced neuronal death. Pretreatment with taurine (0.4 mM and 1.6 mM) decreased UCB-induced apoptotic cell death, which was connected to a reversal of the UCB-mediated increase in [Ca^2+^]_i_ levels [122]. In addition, taurine produced activity against UCB-induced neurotoxicity in a mouse model. Neural apoptotic levels, caspase-3 activity and [Ca^2+^]_i_ were noticeable after the induction of UCB but pretreatment with taurine (7.5 or 15 mg/kg for 4 h) attenuated apoptotic death by downregulating caspase-3 and [Ca^2+^]_i_ [123].

In the auditory system, taurine also attenuated UCB-induced neuronal injury in neonatal guinea pigs [124]. Elevated latencies, interwave intervals and thresholds of auditory brainstem responses and action potential were observed upon the UCB injection. In addition, UCB treatment produced noticeable injury to type I spiral ganglion neurons, their axons, and terminals to cochlear inner hair cells. In the peripheral and central auditory system, five days of taurine treatment significantly ameliorated UCB-mediated electrophysiological anomalies and morphological damage [124].

#### 4.7.3 Angelman syndrome and Fragile X syndrome

In a designed model, taurine (1 g/kg, p.o) produced neuroprotective activity in Ube3a-deficient mice with Angelman syndrome. Treatment with taurine suggestively ameliorated abnormal motor and learning behavior and reestablished postsynaptic density protein-95 levels and ERK phosphorylation [125]. As a genetic disease, Fragile X syndrome is characterized by behavioral complaints and moderate to severe intellectual debilities. The cognitively impaired Fragile X mouse displays a positive response to taurine treatment (0.05% w/v, p.o for 4 weeks) and outcome was outwardly associated with GABAergic action [126,127].

#### 4.7.4 Sleep-wake disorders

The role of caffeine and taurine in sleep-wake activity has been studied in a model utilizing *Drosophila melanogaster*. Taurine treatment elevated sleeping duration, but caffeine attenuated sleep. In addition, cotreatment with caffeine and taurine displayed two differential actions that were dependent on the taurine and caffeine ratio. A high ratio between taurine and caffeine promoted sleep, whereas a low ratio prevented sleep to a larger extent in comparison with an equal amount of caffeine treatment alone. The low doses of taurine-mediated enhancing action of caffeine may provide a justification for the occurrence of both components in energy-enhancing drinks, including Monster^®^ and Red Bull^®^ [128].

#### 4.7.5 Neural tube defects

Taurine has potential activity against neural tube defects. In a study, taurine showed protective activity against glutamate-mediated damage in a hippocampal neuron cell line (HT-22). Treatment with low-dose (0.5 mmol/L) and high-dose (2.0 mmol/L) of taurine produced decreasing in the glutamate-induced apoptosis rate and caspase-3 activation. In addition, taurine protected retinoic acid-induced embryonic neural tube defects in Kunming mice. Taurine (2 g/L solution) treatment significantly attenuated the expression of dishevelled, ras homolog gene family member A, and phosphorylated c-Jun N-terminal kinase in retinoic acid-induced mice. It was suggested that Wnt/planar cell polarity-*c*-Jun N-terminal kinase-pathway may be connected to the protective role of taurine against NTD [18]. Besides, taurine action against neuroinflammation, oxidative stress and excitotoxicity may have the potential to attenuate neural tube defects.

#### 4.7.6 Cerebral palsy

As a potential agent, taurine shows an ameliorating role in preventing cerebral palsy. In a study, Sprague-Dawley rats were treated with a low protein diet daily to establish an intrauterine growth-restricted (IUGR) model from pregnancy to parturition, and 300 mg/kg taurine was added every day to the diet from the 12th day of pregnancy. Taurine supplementation in pregnant rats declined cell apoptosis in brain tissue from neonatal rats with intrauterine growth restriction. The supplement also elevated glial cell line-derived neurotrophic factor (GDNF) expression and reduced caspase-3 expression in the cerebral cortex. In addition, supplementation decreased apoptosis via the GDNF-caspase-3 signaling pathway [129]. In another study utilizing the IUGR model, antenatal administration of taurine promoted cell proliferation and activated GDNF in fetal rat brain. Taurine activated the protein kinase A-cAMP response element-binding protein signaling pathway, increased GDNF, and promoted cell proliferation to counter IUGR-mediated neuronal loss [130].

#### 4.7.7 Attention deficit hyperactivity disorder

In an attention-deficit hyperactivity disorder (ADHD) model, taurine has a potential effect on resting-state fMRI activity. Administration of high dose of taurine (45 mmol/kg, 4 weeks) suggestively reduced serum C-reactive protein in Wistar Kyoto (WKY) rats, but both low-taurine (22.5 mmol/kg, 4 weeks) and high-taurine treatments significantly reduced interleukin (IL)-1β and C-reactive protein in spontaneously hypertensive (SH) rats. In addition, low-taurine administration led to significantly greater horizontal locomotion in WKY and SH rats in comparison with controls. On the other hand, lower horizontal locomotion was observed after high-taurine treatment in SHR. Additionally, a high dose of taurine produced pointedly lower functional connectivity (FC) and mean amplitude of low-frequency fluctuation (mALFF) in the bilateral hippocampus in WKY and SH rats. Remarkably, low-taurine and high-taurine administration lowered the mALFF in rats compared with the SHR control group. According to the study, high-dose taurine treatment possibly ameliorated hyperactive performance in SH rats by modifying the brain's functional signals and attenuating inflammatory cytokines [131].

## 5 Neuropharmacological potential of taurine analogs

Several taurine analogs are potential agents for treating neurological disorders. As an endogenous modulator, γ-l-glutamyltaurine has an effect on excitatory amino-acidergic neurotransmission, in which it prevented the glutamate-evoked elevation in free [Ca^2+^]_i_ as well as kainate-mediated cGMP formation in cerebellar slices [132]. The micronucleus test revealed that γ-l-glutamyltaurine protected mitomycin C-mediated genotoxic action in rat bone marrow cells [133].

Adding a carbon atom to taurine yields homotaurine, an analog of taurine. In 1965, Abbott Laboratories first patented this analog and marketed it as tramiprosate. As a promising drug, homotaurine (Alzhemed™) is also known for treating Alzheimer's disease. Synergistic action was achieved when citicoline combined with homotaurine protected glutamate- and high glucose-treated toxicity in retinal cultures [134]. A very recent study demonstrated its potential protective role against multiple sclerosis [135]. Homotaurine, a safe BBB-permeable GABA_A_-R-specific agonist, attenuated multiple sclerosis pathogenesis in a model study. It has the ability to cross the BBB and improve CD8^+^ and CD4^+^ Treg cell responses and limits Th17- and Th1-mediated CNS inflammation [135]. It prevented amyloid formation and deposition and preserved Aβ in a nonfibrillar form though preferentially binding to soluble Aβ peptide [136]. In addition, homotaurine action is dependent on changes in cortical GABA transmission, displaying a potential role in attenuating cholinergic transmission by modulating inhibitory cortical action [137]. Homotaurine treatment reduced volume loss in the left and right hippocampal tail, left and right fusiform gyrus, and right inferior temporal cortex of patients, which demonstrated its role in improving short-term memory performance. Therefore, homotaurine supplementation in individuals with mild cognitive impairment has a beneficial effect on hippocampus atrophy and episodic memory loss. Further investigation should be performed to elucidate its mechanism of action on brain morphometry [138]. Moreover, this drug also elevated striatal dopamine, which is independent of impulse flow or exocytosis. Inversely, taurine-evoked upsurge in striatal dopamine was dependent on impulse flow [139].

As a prodrug of taurine, N-pivaloyltaurine (a slight variant of taurine) in the brain can produce the same effect as taurine regarding the production of striatal dopamine [140]. N-isopropylamide-2-(1-phenylethyl)aminoethanesulfonic acid hydrochloride, which is also known as TAU-15 and taurepar, displayed neuroprotective action in a brain ischemia model [141]. In ischemic conditions, TAU-15 activated carbohydrate aerobic oxidation, elevated energy metabolism and prevented lipid peroxidation activation. It also restored the antioxidant system, regulated free radical generation and elevated animal survivability by 40%. TAU-15 also displayed an ameliorating effect on compressed spinal cords [142].

Another taurine analog, tauropyrone, a neuroprotective agent, has no activity against convulsions [143]. It protected the oxidation of dopamine in a PD model, and due to its lipophilic nature, it is not functionally TauT-dependent [143]. Another study demonstrated its protective activity against oxygen-glucose deprivation (OGD)-mediated cell damage by ameliorating lactate dehydrogenase production and excitotoxicity [144]. The OGD model was used to study taurine analogs/GABA-T inhibitors such as piperidine-3-sulfinic acid (PSA), 2-aminobenzenesulfonate (ANSA) and 2-(N-acetylamino) cyclohexane sulfonic-acids (TAHS). These analogs were also able to decrease lactate dehydrogenase and glutamate release via reducing GABA metabolism, resulting in an elevation in GABAergic transmission [145,146]. Dzirkale et al. (2011) reported an anti-ethanol effect, in which small doses of tauropyrone decreased ethanol-mediated sleeping time [147].

Acetylhomotaurine derivative, acamprosate (calcium acetylhomotaurinate), are used as the most indicated for treating alcohol abuse. A decline in ethanol self-administration and drinking relapses were observed in both animals and humans after its administration. Collectively, the endogenous taurine system may be a crucial modulator of ethanol on the nervous system and may signify a novel therapeutic avenue for the development of medicines to treat conditions of alcohol abuse as well as alcoholism [148]. In 2004, US-FDA approved acamprosate (Campral™) for treating alcohol dependence. It showed ameliorating activity in alcohol-dependent individuals with bipolar disorder in a prior clinical trial report [149].

Taurine has generally been found in the photoreceptor cell layer of the retina. The deficiency of taurine in mice results in anomalous bipolar cell plasticity and retinal ganglion cell loss [150]. The most promising derivatives, 2- thiomorpholine 1,1-dioxide (TMS), aminoethylmethylsulfone (AEMS) and N-methyl- thiomorpholine 1,1-dioxide (MTMS), are more potent than other cyclic compounds including TAHS and CAHS. They exerted their activity by ATP-mediated Ca^2+^ uptake [151].

2-Phthalimidosulfonamide derivatives of taurine were synthesized and investigated for anticonvulsant activity by Lindén et al.(1983) [152]. According to a study on the structure-activity relationship, having a two-carbon chain in the taurine molecule is vital for better action of these derivatives. In addition, substitutions of the terminal sulfonamide moiety raised the lipophilicity of these derivatives, which helped them cross the BBB, although the activity was reduced when large functional groups were connected to the sulfonamide moiety.

The activity of several taurine derivatives against convulsions in the models of maximum electroshock seizure and pentetetrazole-induced seizure threshold has been found. Derivatives with unsubstituted amides (methylamide, dimethylamide, ethylamide and isopropylamide) displayed almost equal efficiency. However, N-propylamide and N-butylamide were also active, but their potency was low. Several derivatives, such as acetamide, pyridylamide, piperidide, pyrrolinedide, cyclohexylamide, benzylamide and methylbenzylamide derivatives, did not produce action. Although the N-isopropyl derivative (called taltrimide) is available for commercial purposes, it is not permitted for therapeutic use because its anticonvulsive effect has been experimental in animal models, not in clinical studies. In contrast, taltrimide treatment significantly promoted seizures, displaying proconvulsant activity in humans; therefore, its mechanism of action remains unclear [153].

Isoherranen et al. (2003) [154] synthesized novel valproyltaurinamide derivatives that are able to act as mutual prodrugs of valproic acid (VPA) and taurine and act as a hybrid. This study was conducted to obtain a more efficacious form of valproic acid, an antiepileptic drug beneficial against epileptic seizures and free from teratogenicity. Three compounds displayed an adequate profile (VTD > I-VTD and DM-VTD) for averting tonic extension. Following the pharmacokinetics study, a moderate correlation between the brain metabolite N-alkyl-VTD and its activity against convulsions was experimental; nonetheless, no connection was detected with its log P value and teratogenic properties. Neural tube defects were observed in the pattern of VTA > DM-VTD > I-VTD > VTD. In addition, M-VTD appeared to exhibit less teratogenic properties (1% of the live-born fetuses), although the difference was nonsignificant. According to the results, valproyltaurinamide derivatives and anilide groups with small substituents on the N-phenyl ring exhibit action against convulsions. In 2007, Akgul et al. repeated the research of Lindén et al. [152] and found 15 novel 2-phtalimidoethanesulfonamide derivatives with phenyl groups connected to the sulfonamide moiety. Per an initial study, the exchange of the N-isopropyl moiety for an N-phenyl ring in the taltrimide molecule abolished its anticonvulsant activity. However, adding certain groups (nitro, methyl, and chloro) into the N-phenyl ring led to more active compounds in the maximal electroshock seizure test compared to the derivatives that are unsubstituted. Neurotoxicity was observed with a methyl substituent in the N-phenyl ring in the rotarod test [154].

Oja et al. (1983) found 23 taurine derivatives by substituting at the amine and sulfonic acid groups. In a study of 9 dynamic compounds including benzamido, piperidino, phthalimido and phenylsuccinimido derivatives, piperidino variants exhibited the greatest activity in the rotarod test than other compounds [155].

In MPP^+^-induced neurodegeneration in a rat model, several taurine analogs (taurine phosphonate, trimethyltaurine and guanidinoethane sulfonate) produced a protective effect via an extracellular mechanism. These effects occur through GABA_A_ receptors, which was confirmed by administering the GABA receptor ligands bicuculline and muscimol [91].

In leukocytes, taurine acts to trap chlorinated oxidants (HOCl). Upon the chemical reaction, taurine and HOCl produced the lasting compound taurine chloramine (Tau-Cl) [156]. Tau-Cl demonstrated anti-inflammatory activity in activated macrophages [156]. Tau-Cl suggestively reduced lymphocyte proliferation in another study. Tau-Cl inhibited the IL-6, IL-8, and IL-2 in phytohemagglutinin-activated nonadherent leukocytes productions. It also reduced the IL-1β, IL-6, and IL-8 in LPS-activated adherent monocytes productions [157]. At higher concentrations, Tau-Cl downregulated proinflammatory cytokine production, although Tau-Cl's effect on TNF-α production by peripheral blood mononuclear cells from rheumatoid arthritis patients is less adequate than in osteoarthritis patients [158].

The neuropharmacological potential of taurine analogs is summarized in Table 1.

**Table 1:** Chemical structures of taurine analogs, their major effects and their ability to cross the BBB.

Derivatives	Ability to cross BBB	Effects	Investigated in
	+	⁃ Affects emotional arousal ⁃ Exhibits synergistic effects with anxiolytic drugs	[159]
	+	⁃ Activates GABA_A_-R and enhances CD8^+^ and CD4^+^ Treg cell responses ⁃ Limits Th17- and Th1-mediated inflammation in the CNS	[160]
	+	⁃ Increases striatal dopamine concentration	[161]
	+	⁃ Modulates cerebral circulation in ischemic mice	[141]
	–	–	–
	–	–	–
	+	⁃ Increases GABA content	[145]
	–	–	–
	+	⁃ Restores NMDA receptor tone in the glutamate system	[162]
	–	⁃ Stimulates ATP-dependent Ca^2+^ uptake and protects the retina	[163]
	–	⁃ Stimulates ATP-dependent Ca^2+^ uptake and protects the retina	[163]
	–	⁃ Stimulates ATP-dependent Ca^2+^ uptake and protects the retina	[163]
	–	–	–
	–	–	–
	+	⁃ Acts on the depolarization-stimulated release of GABA	[164]
	+	?	[165]
	+	⁃ Inhibits ChE and MAO-B activities	[166]
	–	–	–
	+	⁃ Possesses anticonvulsant properties	[154]

+: Does cross; -: Does not cross; ?: Unknown.

## 6 Clinical study of taurine in neurological disorders

Taurine has been studied clinically in different diseases. The frequency, duration and intensity of muscle cramps were reduced by taurine treatment (2 g per day, p.o) in a recent clinical study on patients with chronic liver disease [167]. Among the different neurological disorders, the clinical study of taurine has been investigated in succinic semialdehyde dehydrogenase (SSADH) deficiency [168] and stroke [169]. SSADH deficiency is a rare autosomal genetic disease affecting a key enzyme in the catabolism of GABA in which patients exhibit different symptoms, including ataxia, hypotonia, communication deficits and intellectual disability. Approximately half of SSADH patients are affected by seizures because the illness is accompanied by disrupted GABA homeostasis. Taurine showed activity against symptoms of SSADH, which was investigated in a single case study on a 2-year-old boy. Taurine (200 mg/kg per day) administration for 12 months ameliorated cognitive deficits [170]. However, taurine (50–200 mg/kg per day) for a year was administered to 18 SSADH-deficient subjects in an open-label study in which major amelioration in adaptive behavior was not seen [168].

The efficiency of taurine in treating stroke has been investigated by several studies on animals [16], but limited investigations have addressed its potential in humans. A relationship between serum taurine levels and stroke risk was not found in a prospective-case study conducted on 14,274 women [171]. Nevertheless, among nonsmokers, there may be a link that deserves more attention, specifically considering that the occurrence of stroke was decreased by 90% in a genetic stroke model (stroke-prone impulsively hypertensive rats) fed a diet rich in taurine [172]. A phase III clinical study was conducted on 10 patients in which all patients with recurrent stroke-like episodes received high-dose taurine (9 g or 12 g per day) for 52 weeks. Supplementation of taurine can efficiently decrease the relapse of stroke-like episodes and elevate taurine modification in mitochondrial tRNALeu (UUR) in a model of encephalopathy, mitochondrial myopathy and lactic acidosis [173].

The anti-neuroinflammatory activity of taurine was evaluated in a recent clinical study conducted on 48 elderly women [174] during which 1.5 g per day of taurine was administered for 14 weeks. In this study, 13 subjects received combined exercise training (CET), and 12 subjects received supplementation with taurine (TAU). Eleven subjects were categorized for exercise training associated with taurine (CET and TAU), and 12 participants were in the control group. In all intervention groups, S100β concentrations were maintained, while a subtle increase in the CG was found. Neuron-specific enolase levels increased significantly only in the taurine group, whereas CET significantly reduced TNF-α, IL-6, and IL-6/IL10, IL-1β/IL-1Ra and TNF-α/IL-10. Taurine also suggestively reduced the ratio between IL-1β and IL-1Ra. The Mini-Mental State Examination score was significantly elevated only in the CET and TAU groups. Following multiple regression analysis, a changing pattern in IL-1β was found, and the Charlson Comorbidity Index was separately associated with changes in S100β. Exercise, along with taurine treatment, produced an anti-inflammatory effect and maintained the integrity of BBB.

## 7 Closing remarks

Currently, analysis of the pathophysiology of neurodegeneration represents a great challenge for scientist [[175], [176], [177]]. Copious scientific articles have reported neuroprotective activity of natural constituents on the various model of neurodegeneration. Therefore, medicines from natural sources might be promising to maintain typical brain health and combat against neurodegenerative diseases [[178], [179], [180], [181], [182]].

**Fig. 2:**
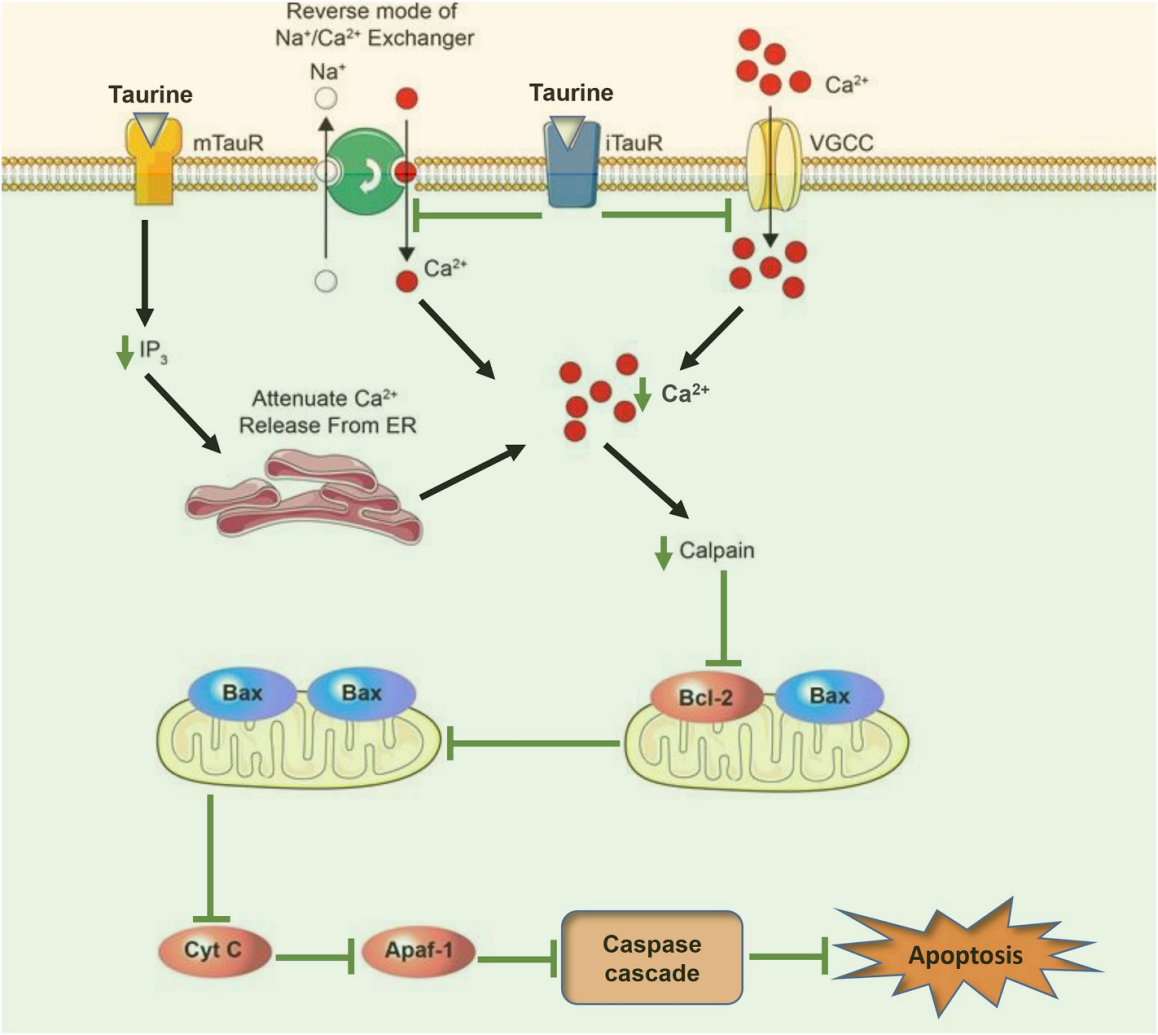
Taurine-mediated neuroprotection against glutamate-induced neuronal death. Taurine activated both ionotropic taurine receptor (iTauR) and metabotropic taurine receptor (mTauR). Upon binding with these receptors, activated iTauR inhibits the reverse mode of sodium/calcium exchangers, as well as inhibits voltage-gated calcium channels (VGCC), leads to decreased intracellular calcium. Activated mTauR also decreases IP3 production, which attenuates the release of calcium from the endoplasmic reticulum (ER). As a result, decreases in intracellular calcium inhibit calpain, which ultimately blocks the conformational changes of Bcl-2 and Bax. This inhibits activation of Bax homodimers that target the mitochondrion-mediated neuronal death cascade.

Exhibiting broad activities, taurine has been considered a potential therapeutic molecule, especially for neurological disorders. According to the prior discussion, it displays extensive inhibitory and regulatory roles that prove its therapeutic efficacy against CNS illnesses. Taurine modulates neurotransmission by acting on several neuroreceptors such as GABA and glutamate and acetylcholine receptors. In addition, it promotes neurogenesis at the preclinical level, and several investigations have reported that it regulates ER stress, energy metabolism, gene expression, quality control processes, Ca^2+^ homeostasis and osmosis. It shows an emergent therapeutic role against several neurological disorders in designed preclinical studies. The protective action of taurine against glutamate-mediated neuronal cell death is portrayed in Fig. 2.

Taurine displayed a protective role against anxiety, depression, neurodegenerative diseases, stroke, epilepsy and diabetic neuropathy. It also protected against trauma- and chemical-mediated neuronal injuries. It has demonstrated ameliorating roles in several models of neurodevelopmental disorders, including Angelman syndrome and Fragile X syndrome, sleep-wake disorders, neural tube defects and attention-deficit hyperactivity disorder. In addition to preclinical studies, taurine played a potential therapeutic role against neuroinflammation, SSADH and stroke at the clinical level. However, more clinical studies necessary to be designed per the preclinical assessment data.

**Fig. 3:**
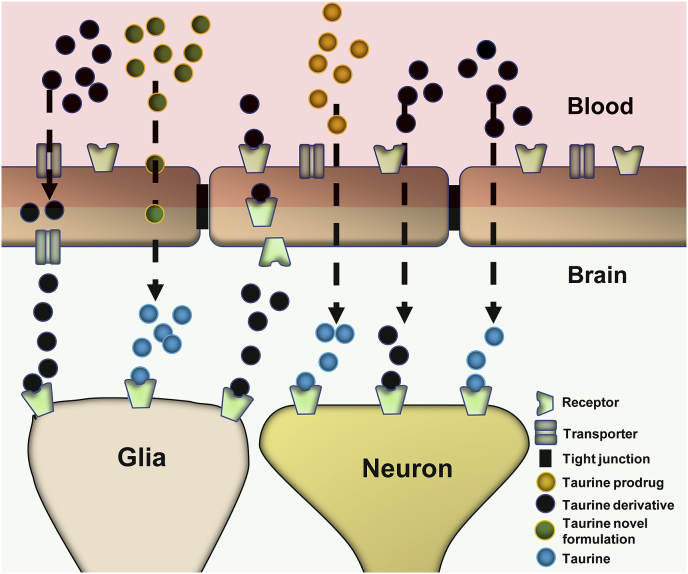
Delivery prospects of taurine for targeting the blood-brain barrier and acting on target sites. This figure proposes that taurine prodrugs may be converted into taurine upon crossing the BBB. Taurine derivatives may be capable of producing action via crossing BBB either forming taurine or their own forms. Novel formulations may be capable of crossing the BBB due to their lipophilic nature and acting on the target site.

Taurine remains to be investigated based on current limitations. The expression of TauT at the BBB under different disease conditions should be investigated because its expression declined instinctively in the BBB of hypertensive rats [183]. In addition, taurine transport through the BBB fails in other disease situations or oxidative stress processes [68]. In ischemic conditions and the acute conditions of PD, the level of taurine was elevated in brain interstitial fluid [184,185], although taurine levels were low in the chronic condition of Parkinson's disease [186]. It is found to defend the CNS in acute disease phases, but if taurine fails to cross the BBB during chronic conditions, there is not a suitable concentration for neuroprotection. Due to the low passive diffusion through the membranes, the absorption of taurine in the gastrointestinal tract is very low. Therefore, the identification of new lipophilic derivatives of taurine that can cross the BBB in disease circumstances and/or increase binding receptors might be a very fascinating area of research. Moreover, novel delivery of taurine should be investigated for treating neurological disorders, especially the bioavailability of these formulations within the brain. Therefore, biopharmaceutical analyses of taurine should be carried out. Fig. 3 shows the delivery prospects of taurine for treating neurological disorders.

As different taurine analogs may potentially treat neurological disorders, a computational study of taurine and its analogs with target molecules should be designed. This would be helpful to find probable targets and analogs of taurine to treat various CNS illnesses. Taurine has been added in different marketed energy drinks, but more studies should be carried out to validate its beneficial health role in the human body. Considering the wider therapeutic potential of taurine, the formulation of taurine-based products for maintaining neuronal health, as well as treating neurological disorders, will be an excellent area of research.

## Contribution statement

MJ and D-KC: Conceived and designed the study.

MJ: Performed the literature review and wrote the manuscript.

SA: Complied the table.

MEH and S-HJ: Performed the literature review.

MJ, I-SK and MSU: Designed and produced the figures.

D-KC: Supervised and handled the correspondence.

All authors read and approved the final manuscript.

## Declarations of interest

The authors declare no competing interests.
